# Pulmonary sequestration with Aspergillus infection presenting as massive hemoptysis and hemothorax with highly elevated carcinoembryonic antigen in pleural effusion that mimics advanced lung malignancy

**DOI:** 10.1186/s40001-021-00519-5

**Published:** 2021-05-25

**Authors:** Wei Luo, Tong-chen Hu, Lincheng Luo, Ya-lun Li

**Affiliations:** 1 Department of Respiratory and Critical Care Medicine, The People’s Hospital of Leshan, Leshan, 614000 Sichuan China; 2 Department of Thoracic Surgery, The People’s Hospital of Leshan, Leshan, 614000 Sichuan China; 3 Department of Respiratory and Critical Care Medicine, West China Hospital of Sichuan University, Chengdu, 610046 Sichuan China

**Keywords:** Pulmonary sequestration, Aspergillus infection, Carcinoembryonic antigen

## Abstract

**Background:**

Pulmonary sequestration (PS) associated with massive hemoptysis, hemothorax, and elevated tumor markers or even lung malignancy has been reported in several studies. These clinical features combined with lung lesions on chest imaging are sometimes hard to differentiate from lung malignancies and often complicate the diagnostic procedure.

**Case presentation:**

A 45-year-old man with PS presented with massive hemoptysis, hemothorax, and extremely elevated carcinoembryonic antigen (CEA) in pleural effusion was initially misdiagnosed with advanced lung carcinoma, but was ultimately diagnosed with PS with Aspergillus infection.

**Conclusions:**

PS is rarely concurrent with lung cancer; most of the time, it is misdiagnosed as a malignancy, especially when presenting with a fungal infection, which could remarkably elevate CEA in pleural effusion.

## Introduction

Pulmonary sequestration (PS) is a congenital pulmonary malformation with an estimated incidence of 2.2–6.6%. It can be defined as a part of the lung that has no normal communication with the bronchial tree and receives blood supply from systemic arteries [1]. In recent decades, there have been several reports of PS associated with massive hemoptysis, hemothorax, and elevated tumor markers such as carcinoembryonic antigen (CEA) or even diagnosed with lung malignancy [2–4]. These clinical features combined with lung lesions on chest imaging are hard to differentiate from lung malignancies and often complicate the diagnostic procedure [5, 6]. Here, we describe a patient with massive hemoptysis, hemothorax, and remarkably elevated pleural fluid CEA who was nearly diagnosed with primary lung malignancy but was ultimately diagnosed with PS with Aspergillus infection. Written consent was obtained from our Institutional Review Board and the patient for this case report.

## Case presentation

**Fig. 1:**
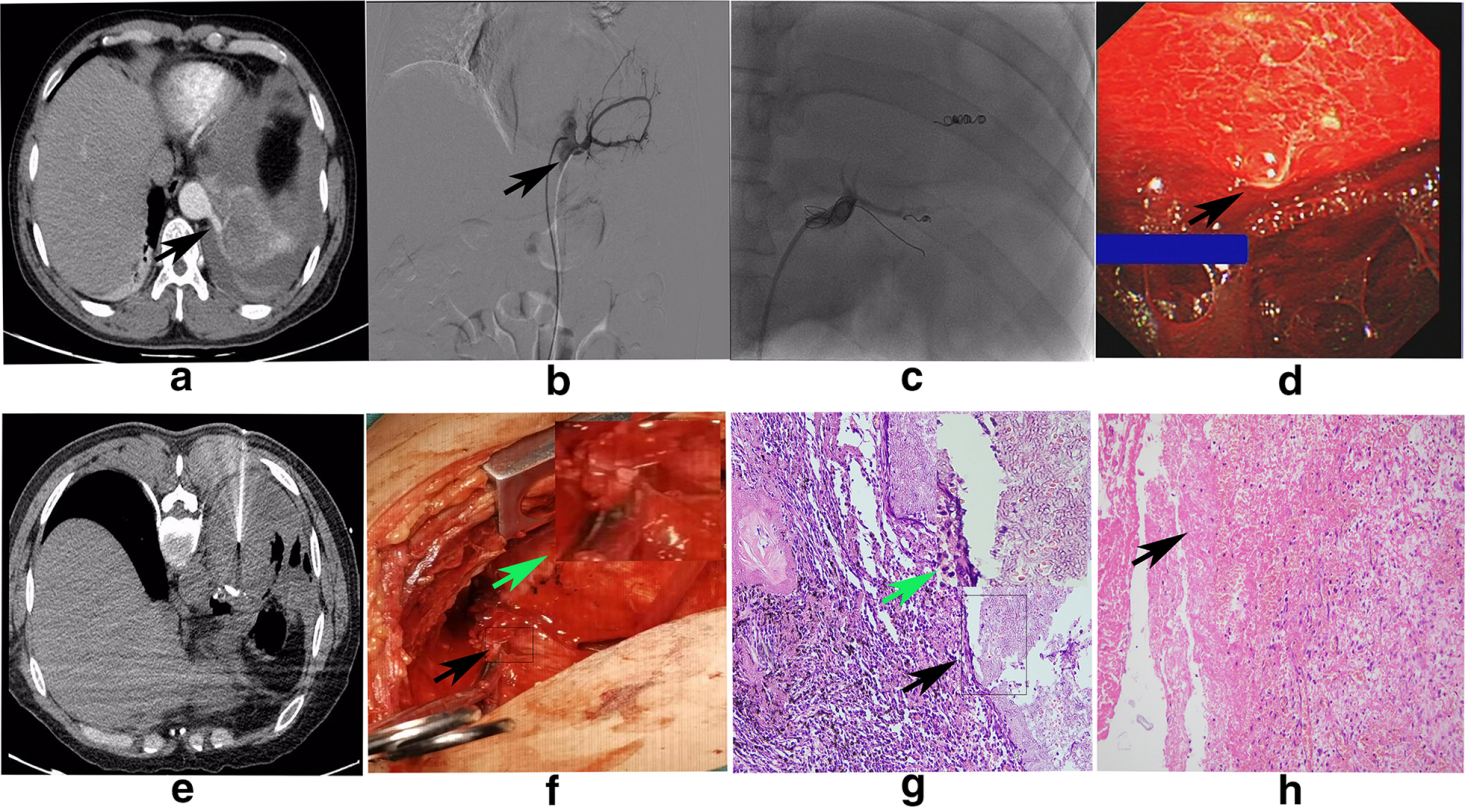
**a** Axial section of thoracic contrast-enhanced CT showing a heterogeneous mass at the posterior basal segment of the left lower lobes with massive pleural effusion (black arrow). **b** Bronchial artery angiography showing an aberrant artery arising from the descending thoracic aorta (black arrow). **c** Aberrant artery embolized with coils and polyvinyl alcohol. **d** Thoracoscopy showing a lesion on the parietal pleura, bloody effusion, and fibrous adhesions (black arrow). **e** CT-guided needle biopsy showing the needle in the mass. **f** Lobectomy showing the aberrant artery as well as the coils (black arrow and rectangle); the right upper corner shows an enlarged view of the artery and coil (green arrow). **g** Septate hyphae in the resected mass with a chronic inflammatory reaction (black arrow and rectangle); the right upper corner shows an enlarged view of the septate hyphae (green arrow). H&E staining, original magnification × 200. **h** Lobectomy showing fibrinoid necrosis with inflammatory cell infiltration. H&E staining, original magnification × 200

A 45-year-old man presented with massive hemoptysis and breathlessness on exertion for 3 days with a history of 30 pack-years of smoking. He had moderate fever but no night sweats, anorexia, or weight loss. The patient did not have any potential medical treatments or relevant pre-existing conditions that may cause these symptoms. The patient had a respiratory rate of 28 breaths/min and oxygen saturation of 91% on ambient air. Chest physical examination revealed mild respiratory distress, obvious percussive dullness, and diminished breath sounds on the left side of the chest. Other physical examination was unremarkable. He had moderate leukocytosis (16,500/µL) and procalcitonin (11.3 mg/L), and serum CEA was normal (2.2 ng/mL). Other laboratory tests were normal. Enhanced chest computed tomography (CT) scans revealed a cystic mass that was mildly enhanced in the left lower lobe supplied by an anomalous artery arising from the descending thoracic aorta with massive pleural effusion (Fig. 1a). Therefore, he was initially diagnosed with PS with inflammatory pleural effusion. Nevertheless, tube thoracostomy revealed hemothorax, while the CEA in the pleural effusion reached 98 ng/ml, and cytological analysis demonstrated that neutrophils were the major type of leukocyte. These findings complicated the diagnosis, such that we could not rule out the possibility of advanced stage lung cancer, so lobectomy was not appropriate in the patient’s condition. Hemoptysis was stopped after bronchial arterial embolism (BAE) with coils and polyvinyl alcohol (Fig. 1b, c). Bronchoscopy and thoracoscopy were performed after BAE. Bronchoscopy was unremarkable, and thoracoscopy showed some lesions on the parietal pleura, bloody effusion and fibrous adhesions (Fig. 1d). However, the histopathology of the pleural lesion only implied chronic inflammation with some necrosis. CT-guided needle biopsy of the retrocardiac mass was performed twice (Fig. 1e). None of the histopathological and cytological examinations suggested malignancy, but only elevated neutrophils and Aspergillus hyphae emerged. Because there was no evidence of malignancy, left lower lobectomy was performed, and the lesion was removed thoroughly (Fig. 1f). Postsurgical histopathology examination confirmed the diagnosis of intralobar pulmonary sequestrations (IPSs) and the existence of septate hyphae as well as some fibrinoid necrosis (Fig. 1g, h). After the surgery, the CEA in the pleural effusion and the temperature returned to normal. The postoperative recovery was unremarkable, and the patient was discharged 7 days later. One month later at outpatient follow-up, the patient showed good recovery, except for slight left chest pain.

## Discussion

We describe a rare case of PS with Aspergillus infection that presented as massive hemoptysis, hemothorax, extremely elevated CEA in pleural effusion and initial radiological findings mimicking advanced lung carcinoma, complicating the therapeutic strategy (not receiving surgery first) and subjecting the patient to repeated biopsies, unnecessary anxiety, and high medical costs. Thus far, only seven cases of lung cancer with PS have been reported [7]. In contrast, up to 21% of PS cases have been misdiagnosed as lung cancer among 2625 cases over a 10-year period in China [8], and Matsuoka et al. [5] also reported that PS tends to be misdiagnosed as lung cancer, especially with high levels of tumor markers.

CEA is one of the most widely used tumor markers for lung cancer and colorectal carcinoma, and can be produced in the epithelium of the respiratory and digestive tracts. CEA participates in the innate immune defense system and has a role in cell adhesion [9]. An oncologic correlation worth mentioning is the relationship between the hemothorax and the remarkably elevated CEA in the pleural effusion in this case. Given the sensitivity and specificity of CEA in diagnosing malignancy [9], the patient was strongly suspected of having advanced lung cancer at the beginning, and we were only able to rule out this suspicion after surgery. There were some reports of PS patients with elevated serum CEA and carbohydrate antigens 19–9 and 125 (CA199, CA125), and most of the CEA corresponding titers were considerably lower (less than 10 ng/mL), except M Sekiya et al. [12], who reported almost five times higher serum CEA levels than normal [4, 5, 10, 11]. Noguchi and Wei Tang separately found elevated serum levels of CEA and CA199 in some patients with allergic bronchopulmonary aspergillosis [12–15]. PS with lung aspergillus has also been reported before [16]. These findings suggest that PS and lung fungal infection could result in elevated tumor markers separately. Whether PS with Aspergillus infection intensifies this phenomenon and whether serum tumor marker concentration and pleural fluid concentration have the same tendency remain unclear. More basic and clinical studies are needed to clarify these questions. Thus far, due to the high misdiagnosis rate of PS with lung cancer, we need to pay more attention to the diagnostic procedure. First, the presence of aberrant vessels to the lung mass is the gold standard of diagnosis of PS, and a careful review of the initial enhanced CT scan is required in the diagnostic procedure. Second, PS patients with Aspergillus infection are not rare [16]; in patients with remarkably elevated tumor marker levels, we definitely need to consider this possibility. Third, in PS patients with recurrent or fatal respiratory symptoms, surgery is the first choice, even if malignancy cannot be ruled out.

## Conclusions

PS could be associated with elevated tumor markers, especially lung Aspergillus infection, which could result in a remarkably increased CEA in either the pleural effusion or serum, leading to a misdiagnosis of advanced lung malignancy.

## Data Availability

All data and materials are available.
